# *N*,*N*′-Bis(2,6-diethyl­phen­yl)acenaphthyl­ene-1,2-diimine

**DOI:** 10.1107/S1600536811052901

**Published:** 2011-12-14

**Authors:** Li Wang, Xuyang Luo, Bo Gao, Qiaolin Wu, Ying Mu

**Affiliations:** a State Key Laboratory of Supramolecular Structure and Materials, School of Chemistry, Jilin University, Changchun 130012, People’s Republic of China; b School of Chemistry, Jilin University, Changchun 130012, People’s Republic of China

## Abstract

The title compound, C_32_H_32_N_2_, has crystallographic twofold rotation symmetry, with two C atoms lying on the rotation axis. The dihedral angle between the substituted benzene ring and the naphthalene ring system is 79.8 (1)°. The crystal structure is stabilized by C—H⋯N inter­actions, which form a chain motif along the *b*-axis direction.

## Related literature

For details and applications of acenaphthenquinone-based Schiff bases and corresponding metal complexes, see: Li *et al.* (2011 ▶); Hagar *et al.* (2010 ▶); Kovach *et al.* (2011 ▶); Oleinik *et al.* (2005 ▶); Ragaini *et al.* (2006 ▶); Rosa *et al.* (2008 ▶); Small *et al.* (2007 ▶); Zhou *et al.* (2008 ▶).
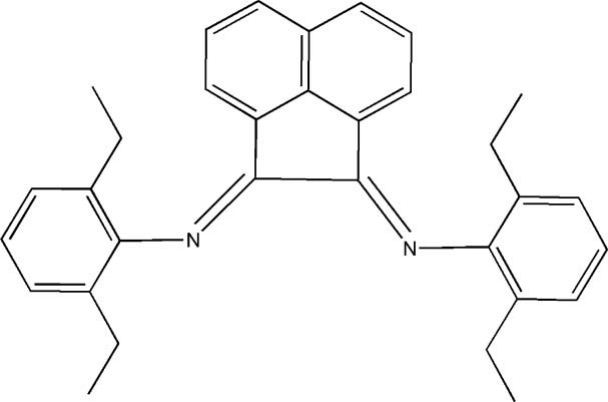


## Experimental

### 

#### Crystal data

C_32_H_32_N_2_
*M* *_r_* = 444.60
Monoclinic, 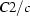

*a* = 13.5134 (12) Å
*b* = 8.6952 (8) Å
*c* = 22.3532 (19) Å
β = 99.413 (1)°
*V* = 2591.2 (4) Å^3^
*Z* = 4
Mo *K*α radiation
μ = 0.07 mm^−1^
*T* = 185 K
0.25 × 0.24 × 0.21 mm

#### Data collection

Bruker SMART APEX CCD diffractometer
Absorption correction: multi-scan (*SADABS*; Bruker, 2001 ▶) *T*_min_ = 0.984, *T*_max_ = 0.986
7599 measured reflections
2538 independent reflections
1842 reflections with *I* > 2σ(*I*)
*R*_int_ = 0.028

#### Refinement

*R*[*F* ^2^ > 2σ(*F* ^2^)] = 0.053
*wR*(*F* ^2^) = 0.151
*S* = 1.02
2538 reflections
157 parameters
H-atom parameters constrained
Δρ_max_ = 0.42 e Å^−3^
Δρ_min_ = −0.19 e Å^−3^

### 

Data collection: *SMART* (Bruker, 1998 ▶); cell refinement: *SAINT* (Bruker, 1998 ▶); data reduction: *SAINT*; program(s) used to solve structure: *SHELXS97* (Sheldrick, 2008 ▶); program(s) used to refine structure: *SHELXL97* (Sheldrick, 2008 ▶); molecular graphics: *SHELXTL* (Sheldrick, 2008 ▶); software used to prepare material for publication: *SHELXTL*.

## Supplementary Material

Crystal structure: contains datablock(s) global, I. DOI: 10.1107/S1600536811052901/fy2033sup1.cif

Structure factors: contains datablock(s) I. DOI: 10.1107/S1600536811052901/fy2033Isup2.hkl

Supplementary material file. DOI: 10.1107/S1600536811052901/fy2033Isup3.cml

Additional supplementary materials:  crystallographic information; 3D view; checkCIF report

## Figures and Tables

**Table 1:** Hydrogen-bond geometry (Å, °)

*D*—H⋯*A*	*D*—H	H⋯*A*	*D*⋯*A*	*D*—H⋯*A*
C4—H4⋯N1^i^	0.95	2.43	3.379 (2)	179

Symmetry code: (i) 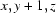.

**Fig. 1.**
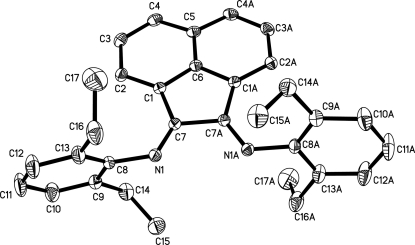
View of the molecule of (I) showing the atom labelling scheme. Displacement ellipsoids are drawn at the 30% probability level. The hydrogen atoms are omitted for clarity. [Symmetry code: (A) -x, y, 0.5 - z.]

**Fig. 2.**
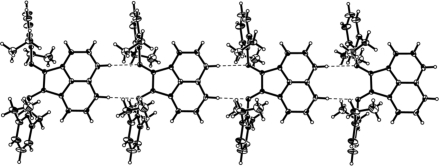
The molecular packing of (I). Hydrogen bonds are indicated by dashed lines.

## supplementary crystallographic information

### Comment

Acenaphthenequinone-based Schiff bases and their metal complexes have been widely synthesized due to their significant applications in catalysis, coordination chemistry and supramolecular assemblies. (Ragaini *et al.*, 2006; Kovach *et al.*, 2011; Small *et al.*, 2007; Rosa *et al.*, 2008; Oleinik *et al.*, 2005; Li *et al.*, 2011; Hagar *et al.*, 2010; Zhou *et al.*, 2008.) As part of our research efforts focused on developing olefin polymerization catalysts, a novel series of Schiff-base derivatives were synthesized and evaluated as high-performance ligand systems. Herein, we report the preparation and crystal structure of new acenaphthenequinone-based Schiff base compound, derived from acenaphthenequinone and 2,6-diethylaniline.

The title molecule, Fig. 1, has crystallographic twofold rotation symmetry. The dihedral angle between the substituted benzene ring and the naphthalene fragment is 79.8 (1)°. Both lengths and angles in the title compound are in normal ranges and are comparable to those found in similar acenaphthenequinone-based Schiff base compounds (Kovach *et al.*, 2011; Rosa *et al.*, 2008).

In the packing of the crystal, there are intermolecular C—H···N interactions, which form one-dimensional chains (Fig. 2 and Table 1).

### Experimental

Acenaphthenequinone (1.35 g, 7.4 mmol) in 65 ml of acetonitrile was heated under reflux for 30 min and then 12 ml of acetic acid was added and heating was continued until the acenaphthenequinone had completely dissolved. To this hot solution, 2.39 g (16 mmol) of 2,6-diethylaniline was added directly and the solution was heated under reflux for a further 1.5 h. It was then cooled to room temperature and the solid filtered off to give a yellow product that was washed with hexane and air dried. Yield 2.73 g (83%). The crystals suitable for X-ray structure determination were obtained by slow solvent evaporation from an ethanolic solution at room temperature.

### Refinement

The C-bound H atoms were positioned geometrically with C—H = 0.95 (aromatic carbon), 0.99 (methylene) and 0.98 (methyl) Å, and allowed to ride on their parent atoms in the riding model approximation with *U*_iso_(H) = 1.2 (1.5 for methyl) *U*_eq_(C).

### Crystal data

**Table:**

C_32_H_32_N_2_	*Z* = 4
*M**_r_* = 444.60	*F*(000) = 952
Monoclinic, *C*2/*c*	*D*_x_ = 1.140 Mg m^−^^3^
Hall symbol: -C 2yc	Mo *K*α radiation, λ = 0.71073 Å
*a* = 13.5134 (12) Å	θ = 3.7–52.1°
*b* = 8.6952 (8) Å	µ = 0.07 mm^−^^1^
*c* = 22.3532 (19) Å	*T* = 185 K
β = 99.413 (1)°	Block, pale yellow
*V* = 2591.2 (4) Å^3^	0.25 × 0.24 × 0.21 mm

### Data collection

**Table:**

Bruker SMART APEX CCD diffractometer	2538 independent reflections
Radiation source: fine-focus sealed tube	1842 reflections with *I* > 2σ(*I*)
graphite	*R*_int_ = 0.028
φ and ω scans	θ_max_ = 26.0°, θ_min_ = 1.9°
Absorption correction: multi-scan (*SADABS*; Bruker, 2001)	*h* = −16→14
*T*_min_ = 0.984, *T*_max_ = 0.986	*k* = −10→10
7599 measured reflections	*l* = −27→24

### Refinement

**Table:**

Refinement on *F*^2^	Primary atom site location: structure-invariant direct methods
Least-squares matrix: full	Secondary atom site location: difference Fourier map
*R*[*F*^2^ > 2σ(*F*^2^)] = 0.053	Hydrogen site location: inferred from neighbouring sites
*wR*(*F*^2^) = 0.151	H-atom parameters constrained
*S* = 1.02	*w* = 1/[σ^2^(*F*_o_^2^) + (0.0768*P*)^2^ + 1.7702*P*] where *P* = (*F*_o_^2^ + 2*F*_c_^2^)/3
2538 reflections	(Δ/σ)_max_ < 0.001
157 parameters	Δρ_max_ = 0.42 e Å^−^^3^
0 restraints	Δρ_min_ = −0.19 e Å^−^^3^

### Special details

**Table:**

Experimental. ^1^H NMR (300 MHz, CDCl_3_, 298 K) δ (p.p.m.): 1.13 (t, *J*_H—H_ = 9.0 Hz, 12H, CH_2_CH_3_), 2.49 (m, 4H, CH_2_CH_3_), 2.60 (m, 4H, CH_2_CH_3_), 6.71 (d, 2H), 7.22 (m, 6H), 7.37 (t, 2H), 7.87 (d, 2H). ^13^C NMR (75 MHz, CDCl_3_, 298 K) δ (p.p.m.): 8.74, 19.58, 117.77, 118.81, 121.28, 122.94, 123.68, 124.41, 125.55, 125.86, 135.46, 143.43, 155.61 p.p.m..
Geometry. All e.s.d.'s (except the e.s.d. in the dihedral angle between two l.s. planes) are estimated using the full covariance matrix. The cell e.s.d.'s are taken into account individually in the estimation of e.s.d.'s in distances, angles and torsion angles; correlations between e.s.d.'s in cell parameters are only used when they are defined by crystal symmetry. An approximate (isotropic) treatment of cell e.s.d.'s is used for estimating e.s.d.'s involving l.s. planes.
Refinement. Refinement of *F*^2^ against ALL reflections. The weighted *R*-factor *wR* and goodness of fit *S* are based on *F*^2^, conventional *R*-factors *R* are based on *F*, with *F* set to zero for negative *F*^2^. The threshold expression of *F*^2^ > σ(*F*^2^) is used only for calculating *R*-factors(gt) *etc*. and is not relevant to the choice of reflections for refinement. *R*-factors based on *F*^2^ are statistically about twice as large as those based on *F*, and *R*- factors based on ALL data will be even larger.

### Fractional atomic coordinates and isotropic or equivalent isotropic displacement parameters (Å^2^)

**Table:**

	*x*	*y*	*z*	*U*_iso_*/*U*_eq_	
C1	0.48604 (12)	1.10939 (19)	0.30114 (7)	0.0252 (4)	
C2	0.47262 (13)	1.1829 (2)	0.35360 (8)	0.0307 (4)	
H2	0.4629	1.1260	0.3884	0.037*	
C3	0.47366 (14)	1.3456 (2)	0.35439 (9)	0.0358 (5)	
H3	0.4654	1.3973	0.3907	0.043*	
C4	0.48623 (14)	1.4314 (2)	0.30468 (9)	0.0346 (5)	
H4	0.4857	1.5405	0.3070	0.041*	
C5	0.5000	1.3587 (3)	0.2500	0.0296 (6)	
C6	0.5000	1.1977 (3)	0.2500	0.0259 (5)	
C7	0.49133 (12)	0.94636 (19)	0.28284 (7)	0.0244 (4)	
C8	0.46314 (14)	0.8115 (2)	0.37023 (8)	0.0290 (4)	
C9	0.36277 (14)	0.8239 (2)	0.37853 (8)	0.0353 (5)	
C10	0.34091 (17)	0.7996 (3)	0.43645 (10)	0.0531 (6)	
H10	0.2735	0.8076	0.4432	0.064*	
C11	0.41560 (19)	0.7640 (3)	0.48439 (10)	0.0664 (8)	
H11	0.3997	0.7510	0.5239	0.080*	
C12	0.51299 (19)	0.7475 (3)	0.47479 (10)	0.0622 (7)	
H12	0.5635	0.7204	0.5078	0.075*	
C13	0.53893 (15)	0.7696 (3)	0.41798 (9)	0.0426 (5)	
C14	0.28009 (15)	0.8561 (3)	0.32569 (9)	0.0446 (5)	
H14A	0.2972	0.9501	0.3046	0.054*	
H14B	0.2170	0.8764	0.3415	0.054*	
C15	0.26253 (17)	0.7249 (3)	0.28009 (10)	0.0542 (6)	
H15A	0.3251	0.7018	0.2651	0.081*	
H15B	0.2110	0.7550	0.2460	0.081*	
H15C	0.2402	0.6334	0.2998	0.081*	
C16	0.64667 (17)	0.7500 (3)	0.40762 (10)	0.0571 (7)	
H16A	0.6811	0.6753	0.4374	0.068*	
H16B	0.6466	0.7075	0.3665	0.068*	
C17	0.7033 (2)	0.8979 (4)	0.41362 (14)	0.0786 (9)	
H17A	0.6698	0.9720	0.3840	0.118*	
H17B	0.7718	0.8800	0.4061	0.118*	
H17C	0.7054	0.9388	0.4547	0.118*	
N1	0.48669 (11)	0.81965 (16)	0.31083 (6)	0.0273 (4)	

### Atomic displacement parameters (Å^2^)

**Table:**

	*U*^11^	*U*^22^	*U*^33^	*U*^12^	*U*^13^	*U*^23^
C1	0.0249 (9)	0.0236 (9)	0.0262 (9)	0.0001 (7)	0.0019 (7)	−0.0007 (7)
C2	0.0353 (10)	0.0303 (10)	0.0267 (10)	0.0014 (7)	0.0054 (8)	−0.0028 (8)
C3	0.0419 (11)	0.0314 (11)	0.0338 (11)	0.0024 (8)	0.0058 (8)	−0.0104 (8)
C4	0.0384 (11)	0.0214 (9)	0.0427 (12)	0.0017 (7)	0.0030 (8)	−0.0066 (8)
C5	0.0284 (13)	0.0230 (13)	0.0359 (15)	0.000	0.0006 (11)	0.000
C6	0.0236 (12)	0.0251 (13)	0.0281 (13)	0.000	0.0017 (10)	0.000
C7	0.0247 (8)	0.0239 (9)	0.0240 (9)	0.0000 (7)	0.0024 (7)	−0.0012 (7)
C8	0.0383 (10)	0.0267 (9)	0.0230 (9)	−0.0045 (7)	0.0077 (7)	−0.0004 (7)
C9	0.0373 (11)	0.0386 (11)	0.0308 (10)	−0.0032 (8)	0.0080 (8)	−0.0010 (8)
C10	0.0409 (12)	0.0832 (18)	0.0377 (12)	−0.0097 (11)	0.0141 (10)	−0.0014 (11)
C11	0.0583 (16)	0.117 (2)	0.0267 (12)	−0.0145 (14)	0.0150 (11)	0.0090 (13)
C12	0.0520 (15)	0.104 (2)	0.0286 (12)	−0.0064 (13)	0.0007 (10)	0.0142 (12)
C13	0.0409 (12)	0.0584 (14)	0.0282 (11)	−0.0055 (9)	0.0053 (9)	0.0060 (9)
C14	0.0333 (11)	0.0582 (14)	0.0427 (12)	0.0037 (9)	0.0069 (9)	0.0033 (10)
C15	0.0395 (12)	0.0708 (16)	0.0486 (14)	−0.0116 (11)	−0.0040 (10)	−0.0032 (12)
C16	0.0445 (13)	0.0889 (19)	0.0357 (12)	0.0050 (12)	0.0002 (10)	0.0137 (12)
C17	0.0503 (15)	0.098 (2)	0.086 (2)	−0.0047 (15)	0.0064 (14)	0.0121 (17)
N1	0.0327 (8)	0.0243 (8)	0.0248 (8)	0.0001 (6)	0.0043 (6)	0.0004 (6)

### Geometric parameters (Å, °)

**Table:**

C1—C2	1.373 (2)	C10—C11	1.382 (3)
C1—C6	1.415 (2)	C10—H10	0.9500
C1—C7	1.480 (2)	C11—C12	1.375 (3)
C2—C3	1.414 (3)	C11—H11	0.9500
C2—H2	0.9500	C12—C13	1.385 (3)
C3—C4	1.372 (3)	C12—H12	0.9500
C3—H3	0.9500	C13—C16	1.521 (3)
C4—C5	1.415 (2)	C14—C15	1.522 (3)
C4—H4	0.9500	C14—H14A	0.9900
C5—C6	1.401 (3)	C14—H14B	0.9900
C5—C4^i^	1.415 (2)	C15—H15A	0.9800
C6—C1^i^	1.415 (2)	C15—H15B	0.9800
C7—N1	1.274 (2)	C15—H15C	0.9800
C7—C7^i^	1.524 (3)	C16—C17	1.491 (4)
C8—C13	1.401 (3)	C16—H16A	0.9900
C8—C9	1.403 (3)	C16—H16B	0.9900
C8—N1	1.417 (2)	C17—H17A	0.9800
C9—C10	1.390 (3)	C17—H17B	0.9800
C9—C14	1.513 (3)	C17—H17C	0.9800
			
C2—C1—C6	119.40 (17)	C10—C11—H11	120.0
C2—C1—C7	134.50 (16)	C11—C12—C13	121.4 (2)
C6—C1—C7	106.10 (15)	C11—C12—H12	119.3
C1—C2—C3	118.29 (17)	C13—C12—H12	119.3
C1—C2—H2	120.9	C12—C13—C8	118.13 (19)
C3—C2—H2	120.9	C12—C13—C16	121.09 (19)
C4—C3—C2	122.42 (17)	C8—C13—C16	120.78 (17)
C4—C3—H3	118.8	C9—C14—C15	113.50 (18)
C2—C3—H3	118.8	C9—C14—H14A	108.9
C3—C4—C5	120.52 (18)	C15—C14—H14A	108.9
C3—C4—H4	119.7	C9—C14—H14B	108.9
C5—C4—H4	119.7	C15—C14—H14B	108.9
C6—C5—C4	116.53 (12)	H14A—C14—H14B	107.7
C6—C5—C4^i^	116.53 (12)	C14—C15—H15A	109.5
C4—C5—C4^i^	126.9 (2)	C14—C15—H15B	109.5
C5—C6—C1^i^	122.85 (11)	H15A—C15—H15B	109.5
C5—C6—C1	122.85 (11)	C14—C15—H15C	109.5
C1^i^—C6—C1	114.3 (2)	H15A—C15—H15C	109.5
N1—C7—C1	133.16 (15)	H15B—C15—H15C	109.5
N1—C7—C7^i^	120.06 (10)	C17—C16—C13	112.3 (2)
C1—C7—C7^i^	106.75 (9)	C17—C16—H16A	109.1
C13—C8—C9	121.42 (16)	C13—C16—H16A	109.1
C13—C8—N1	118.61 (16)	C17—C16—H16B	109.1
C9—C8—N1	119.36 (16)	C13—C16—H16B	109.1
C10—C9—C8	117.95 (18)	H16A—C16—H16B	107.9
C10—C9—C14	120.84 (18)	C16—C17—H17A	109.5
C8—C9—C14	121.16 (16)	C16—C17—H17B	109.5
C11—C10—C9	121.1 (2)	H17A—C17—H17B	109.5
C11—C10—H10	119.4	C16—C17—H17C	109.5
C9—C10—H10	119.4	H17A—C17—H17C	109.5
C12—C11—C10	119.9 (2)	H17B—C17—H17C	109.5
C12—C11—H11	120.0	C7—N1—C8	122.72 (15)
			
C6—C1—C2—C3	−0.2 (2)	C13—C8—C9—C14	−174.83 (19)
C7—C1—C2—C3	178.84 (18)	N1—C8—C9—C14	−3.9 (3)
C1—C2—C3—C4	0.9 (3)	C8—C9—C10—C11	−0.1 (3)
C2—C3—C4—C5	−0.7 (3)	C14—C9—C10—C11	177.3 (2)
C3—C4—C5—C6	−0.04 (19)	C9—C10—C11—C12	−2.0 (4)
C3—C4—C5—C4^i^	179.96 (19)	C10—C11—C12—C13	1.6 (4)
C4—C5—C6—C1^i^	−179.32 (11)	C11—C12—C13—C8	0.8 (4)
C4^i^—C5—C6—C1^i^	0.68 (11)	C11—C12—C13—C16	−179.5 (2)
C4—C5—C6—C1	0.68 (11)	C9—C8—C13—C12	−2.9 (3)
C4^i^—C5—C6—C1	−179.32 (11)	N1—C8—C13—C12	−173.9 (2)
C2—C1—C6—C5	−0.55 (17)	C9—C8—C13—C16	177.40 (19)
C7—C1—C6—C5	−179.85 (8)	N1—C8—C13—C16	6.4 (3)
C2—C1—C6—C1^i^	179.45 (18)	C10—C9—C14—C15	−110.2 (2)
C7—C1—C6—C1^i^	0.15 (8)	C8—C9—C14—C15	67.1 (3)
C2—C1—C7—N1	−1.4 (3)	C12—C13—C16—C17	−93.2 (3)
C6—C1—C7—N1	177.72 (17)	C8—C13—C16—C17	86.5 (3)
C2—C1—C7—C7^i^	−179.52 (19)	C1—C7—N1—C8	7.1 (3)
C6—C1—C7—C7^i^	−0.4 (2)	C7^i^—C7—N1—C8	−175.03 (17)
C13—C8—C9—C10	2.6 (3)	C13—C8—N1—C7	−109.9 (2)
N1—C8—C9—C10	173.49 (18)	C9—C8—N1—C7	78.9 (2)

Symmetry codes: (i) −*x*+1, *y*, −*z*+1/2.

### Hydrogen-bond geometry (Å, °)

**Table:**

*D*—H···*A*	*D*—H	H···*A*	*D*···*A*	*D*—H···*A*
C15—H15A···N1	0.98	2.48	3.106 (3)	122.
C16—H16B···N1	0.99	2.51	2.862 (3)	101.
C4—H4···N1^ii^	0.95	2.43	3.379 (2)	179.

Symmetry codes: (ii) *x*, *y*+1, *z*.
